# Intracranial hemorrhagic vasculitis in a child with varicella zoster virus infection: a case report

**DOI:** 10.3389/fmed.2025.1581803

**Published:** 2025-07-10

**Authors:** Saket Satyasham Toshniwal, Jiwan Kinkar, Anand Loya, Palash Sandeep Kotak, Sourya Acharya, Anil Wanjari

**Affiliations:** ^1^Department of General Medicine, Jawaharlal Nehru Medical College, Datta Meghe Institute of Higher Education and Research, Wardha, India; ^2^Department of Neurology, Jawaharlal Nehru Medical College, Datta Meghe Institute of Higher Education and Research, Wardha, India; ^3^Department of General Medicine, Pad. DY Patil Hospital, Navi Mumbai, India

**Keywords:** stroke, child, vasculitic stroke, vasculitis, infection, varicella zoster virus

## Abstract

**Background:**

Varicella zoster virus (VZV) infection is a common viral illness in children, typically manifesting as chickenpox and having a benign, self-limiting course. However, severe neurological complications—such as vasculitis resulting in intracranial hemorrhage—although rare, can occur and are associated with significant morbidity and mortality.

**Case presentation:**

We present the case of a previously healthy 7-year-old boy who developed intracranial hemorrhagic vasculitis 14 days after a primary VZV infection. The child experienced sudden-onset seizures and altered sensorium. Neuroimaging showed an intracranial hemorrhage with evidence of cerebral vasculitis. Laboratory tests, cerebrospinal fluid (CSF) analysis, and serological studies confirmed a recent VZV infection. The patient was treated with antiviral therapy and corticosteroids, resulting in improvement.

**Discussion:**

This case illustrates a rare but severe vascular complication of VZV infection in children. The pathophysiology is viral-induced inflammation of the vessels, leading to vessel wall damage, necrosis, and possible hemorrhage. Early detection through clinical suspicion, imaging, and CSF examination is essential for starting early treatment, which can change the course of the disease and improve outcomes.

**Conclusion:**

Intracranial hemorrhagic vasculitis is an uncommon but serious complication of VZV infection in children. This case highlights the need to consider post-infectious vasculitis in the differential diagnosis of any child with acute neurological symptoms and a recent history of chickenpox to avoid delays in diagnosis and management.

## Introduction

Varicella zoster virus (VZV), a human alpha-herpesvirus that is neurotropic, is the etiologic agent of primary varicella (chickenpox) and herpes zoster (shingles) after reactivation from latency in cranial nerves or dorsal root ganglia. Although primary VZV infection is usually self-limiting in immunocompetent children, it may sometimes result in severe neurological complications. Of these, VZV-associated vasculopathy is an accepted but underdiagnosed condition with inflammatory cerebral arterial involvement that results in ischemic or hemorrhagic stroke, especially in pediatric groups (1, 2).

Intracranial hemorrhagic vasculitis due to VZV infection is still extremely rare, with few reports detailing its occurrence in children. The pathogenesis includes direct viral invasion of cerebral arterial walls, immune-mediated vascular damage, and resultant vessel wall necrosis or rupture. Clinical presentations can be extremely varied, ranging from transient ischemic attacks to fulminant intracerebral hemorrhage (1, 2).

The current literature emphasizes the importance of early diagnosis and recognition of VZV vasculopathy, particularly in light of the fact that antiviral treatments are available that have the potential to substantially alter the course of the disease. MRI and CSF examinations, in combination with VZV DNA or antibody detection, continue to play a central role in making the diagnosis (3, 4).

In this regard, we present a severe and unusual case of intracranial hemorrhagic vasculitis in a previously healthy child following primary varicella infection. This case highlights the importance of increased clinical suspicion and prompt intervention in pediatric patients with acute neurological symptoms in the context of recent VZV infection.

## Case presentation

A previously healthy 7-year-old boy was brought to the emergency department by his mother with a history of irritability, two episodes of vomiting, and agitation that had started earlier that morning. The mother reported that the child had appeared well the night before. Case summary has been shown in Table 1.

**Table 1:** Case summary.

Sequential events	Interpretation with relevant data
1) Case presentation (brief history and symptoms)	A 7-year-old previously healthy boy was brought to the emergency department by his mother with a history of irritability, two episodes of vomiting, and agitation that started earlier that morning. Two weeks before admission, the child had been diagnosed with varicella (chickenpox) at a nearby primary healthcare center, with a fever and a vesicular rash primarily on his trunk and upper limbs.
2) Clinical examination	The child was febrile with an axillary temperature of 38.5°C. He appeared anxious, highly irritable, and had a Glasgow Coma Scale (GCS) score of 13. Neurological examination revealed signs of meningeal irritation, including neck rigidity, photophobia, a positive Kernig’s sign, and a positive Brudzinski’s sign. Deep tendon reflexes were brisk with bilateral extensor plantar responses, but no other focal neurological deficits were noted. Cardiovascular and respiratory tests were benign. There were multiple small, healing vesicular lesions on the trunk and the upper limbs, which were consistent with acute varicella infection.
3) Investigations	The first laboratory analyses revealed peripheral smear neutrophilic leukocytosis and increased acute inflammatory markers. The basic metabolic panel was within normal parameters. Cerebrospinal fluid (CSF) analysis revealed increased protein content and lymphocytic pleocytosis. The CSF polymerase chain reaction (PCR) tested positive for varicella zoster virus (VZV).
4) Radiological investigation 5) Diagnosis	MRI of the brain with MR angiography revealed vasculitic hemorrhage in the left cerebellar hemisphere bordering the left middle cerebellar peduncle. MR angiography revealed irregular stenotic cerebral vessels, particularly involving the middle cerebral artery, and a hypodense region suggestive of hemorrhage (Figures 1–3). The MR spectroscopy results were consistent with a vasculitic hemorrhagic stroke (Figure 4). Intracranial vasculitic hemorrhage secondary to a varicella zoster virus infection
6) Treatment 7) Outcome	IV acyclovir at a dose of 30 mg/kg/day. IV methylprednisolone at a dose of 2 mg/kg/day. Low-dose Aspirin (5 mg/kg/day) for the prevention of vasculitis-related thrombotic events. Intravenous supportive care with fluids, antipyretics, analgesics, and other symptomatic treatment. The child’s condition improved progressively over the next few days. His headaches disappeared, irritability reduced, and he regained more alertness and interaction. One week later, a follow-up MRI revealed no deterioration of the hemorrhage and resolution of focal vasculitis without any new infarcts or hemorrhage.

## Background and relevant history

Two weeks before admission, the child had been diagnosed with varicella (chickenpox) at a nearby primary healthcare center, with fever and a vesicular rash primarily on his upper limbs and trunk. He was treated symptomatically and referred to a higher-level center, but when his fever resolved, no follow-up consultation was undertaken. There was no history of prior vascular disease, immunosuppression, or other major medical conditions.

## On presentation (current status)

In the emergency department, the child was febrile, with an axillary temperature of 38.5°C. He appeared anxious and highly irritable, with a Glasgow Coma Scale (GCS) score of 13. A neurological examination revealed signs of meningeal irritation, including neck rigidity, photophobia, and positive Kernig’s and Brudzinski’s signs. His deep tendon reflexes were brisk, and bilateral extensor plantar responses were noted, though no other focal neurological deficits were present. Cardiovascular and respiratory tests were benign. Multiple small, healing vesicular lesions were observed on the trunk and the upper limbs, which were in line with recent acute varicella infection.

## Laboratory tests

The first laboratory analyses revealed a peripheral smear with neutrophilic leukocytosis and increased acute inflammatory markers. The basic metabolic panel was within normal parameters.

Cerebrospinal fluid (CSF) analysis revealed increased protein content and lymphocytic pleocytosis. The CSF polymerase chain reaction (PCR) tested positive for the varicella zoster virus (VZV). These results are presented in Table 2.

**Table 2:** Cerebrospinal fluid (CSF) analysis findings on admission.

CSF parameters	Results
Opening pressure	25 cm of water
Color	Blood tinged
Red blood cells	2,000 cells/microliter
White blood cells	90 cells/microliter
Protein	300 mg/dL
Glucose	30 mg/dL
VZV PCR	Positive
Cytology	Lymphocytes present

This table summarizes the CSF parameters obtained through lumbar puncture in a patient presenting with irritability, fever, and signs of meningeal irritation. The analysis revealed a raised opening pressure, blood-tinged appearance, elevated red and white blood cell counts with lymphocytic predominance, markedly raised protein levels, and low glucose concentration. Crucially, the varicella zoster virus (VZV) PCR test was positive, confirming viral meningoencephalitis. Cytology showed lymphocytes, supporting the viral etiology.

## Radiological and diagnostic studies

**Figure 1:**
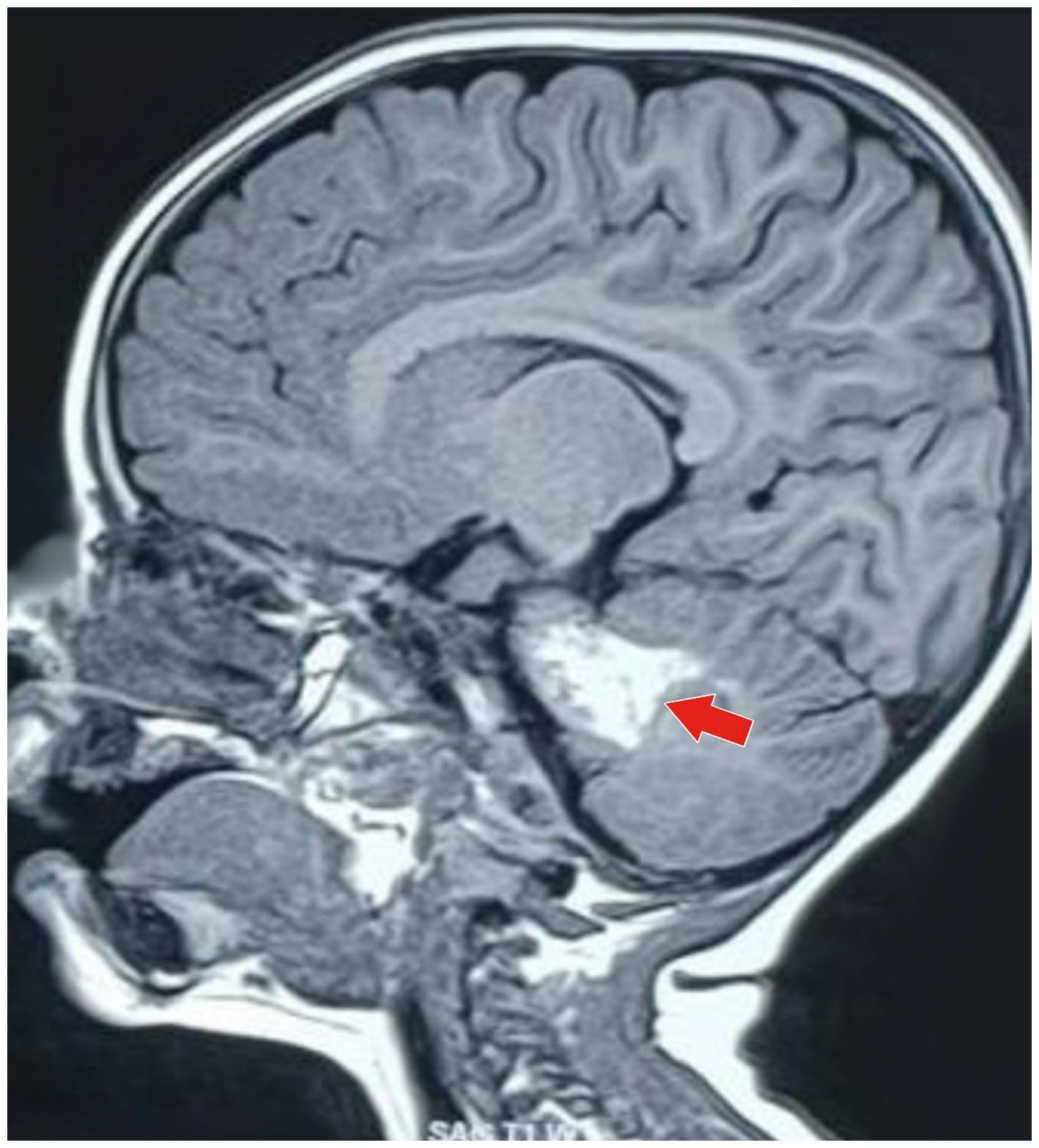
MRI of the brain (sagittal T1-weighted image) showing a well-defined hyperintense lesion (marked by the red arrow), which is typical of an acute parenchymal hemorrhage. The lesion is in the anterior part of the left cerebellar hemisphere, bordering the left middle cerebellar peduncle. It is approximately 3.5 cm (anteroposterior) × 1.8 cm (craniocaudal) × 1.8 cm (transverse). This imaging appearance is typical of a localized intracerebellar hemorrhagic event.

**Figure 2:**
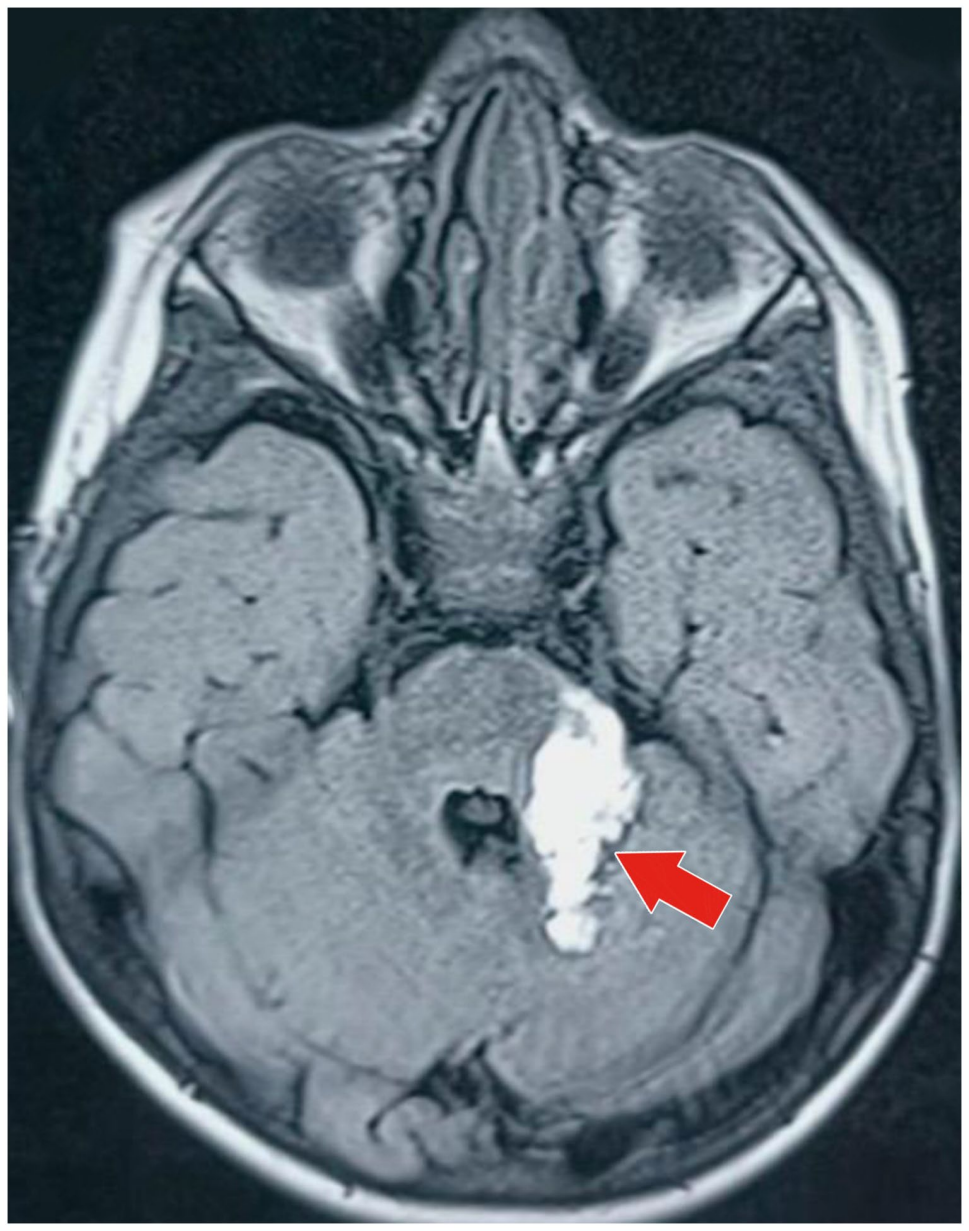
MRI of the brain (axial T1-weighted image) of the same hyperintense lesion (marked by the red arrow) depicting acute parenchymal hemorrhage in the anterior part of the left cerebellar hemisphere, adjacent to the left middle cerebellar peduncle. The lesion is approximately 3.5 cm × 1.8 cm × 1.8 cm in size, with minimal surrounding residual edematous changes seen in the superior part of the left cerebellar hemisphere, reflecting local inflammatory response secondary to vasculitic hemorrhage.

**Figure 3:**
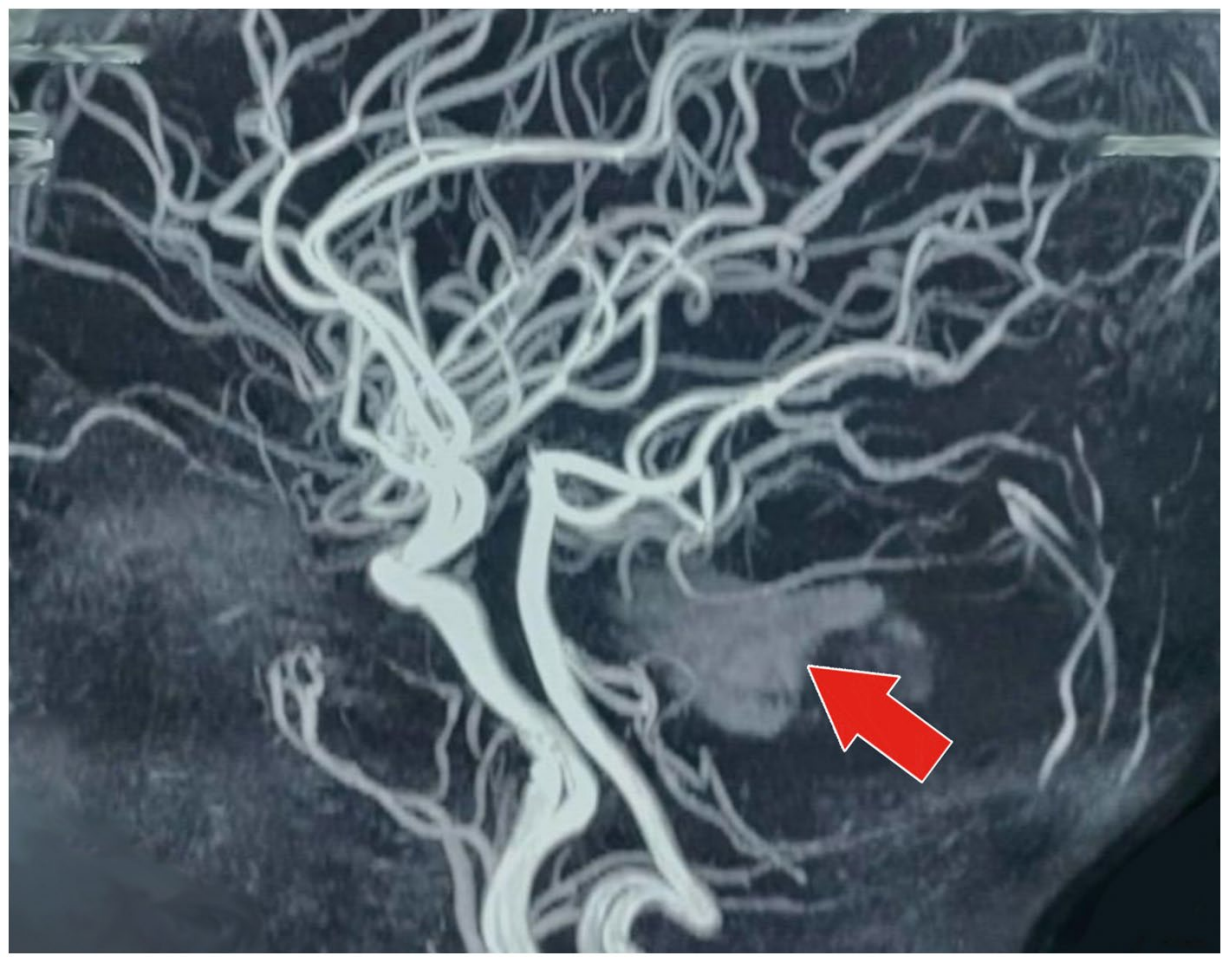
MR angiography of the brain (sagittal projection) showing irregular, stenotic segments of the cerebral vessels, especially the middle cerebral artery (depicted by the red arrow). The picture also demonstrates a hypodense area next to the involved vessels, which corresponds to the location of the hemorrhage. These changes in the vessels are strongly indicative of VZV-related cerebral vasculitis, which caused the intracranial hemorrhagic event.

**Figure 4:**
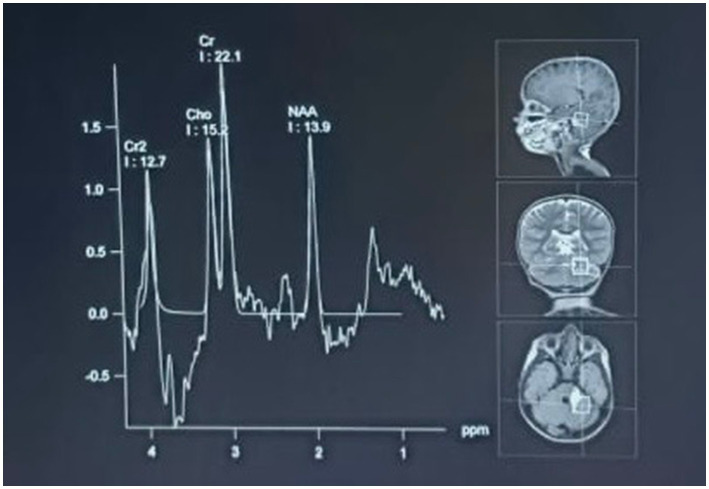
MR spectroscopy of the hyperintense lesion showed a significantly decreased N-acetylaspartate (NAA) peak with increased choline and creatine peaks. This metabolic profile is consistent with a vasculitic hemorrhagic stroke with parenchymal injury and reactive gliosis. The spectroscopic results are consistent with the diagnosis of inflammatory vascular injury due to viral infection.

Based on the neurological examination and recent VZV infection, neuroimaging was conducted. An MRI of the brain with MR angiography revealed vasculitic hemorrhage in the left cerebellar hemisphere bordering the left middle cerebellar peduncle. MR angiography revealed irregular, stenotic cerebral vessels, particularly involving the middle cerebral artery, and a hypodense region suggestive of hemorrhage (Figures 1–3). The MR spectroscopy results were in line with a vasculitic hemorrhagic stroke (Figure 4).

## Diagnosis and management

Based on the clinical presentation, the patient’s recent history of VZV infection, CSF findings, and neuroimaging results, a diagnosis of VZV-associated intracranial vasculitic hemorrhage was established, which indicated a hemorrhagic transformation of ischemic vasculitis secondary to a varicella zoster virus infection.

The child was admitted to the pediatric intensive care unit for close monitoring of the nervous system and management of the hemorrhage secondary to vasculitis. Treatment included:

- Intravenous acyclovir at a dose of 30 mg/kg/day.
- Intravenous methylprednisolone at a dose of 2 mg/kg/day.
- Low-dose Aspirin (5 mg/kg/day) for the prevention of vasculitis-related thrombotic events after stabilization and continued at discharge.
- Supportive intravenous care with fluids, antipyretics, analgesics, and other symptomatic treatments.

## Clinical course and outcome

The child’s condition progressively improved over the next few days. His headaches disappeared, his irritability reduced, and he regained more alertness and interaction. One week later, a follow-up MRI revealed no progression of the hemorrhage and resolution of focal vasculitis without any new infarcts or hemorrhages.

The child was discharged on the 10th day of his hospital stay on a tapering dose of oral corticosteroids and was continued on oral acyclovir for 2 more weeks. Low-dose Aspirin was prescribed for 6 months to prevent the recurrence of thrombotic events. He was referred to the outpatient clinics of Neurology and Infectious Diseases for follow-up.

## Discussion

Intracranial hemorrhagic vasculitis secondary to primary varicella zoster virus (VZV) infection is an extremely uncommon but potentially life-altering complication in children. Although VZV is typically found to be related to a self-limiting, benign illness in immunocompetent children, neurological complications, most notably vasculopathy, may ensue and are characterized by substantial morbidity and mortality. Hemorrhagic stroke as a manifestation of VZV-associated vasculitis is also rarely reported; thus, this case is of particular importance since it highlights a rare and dangerous vascular complication that develops during the post-varicella phase (5).

### Comparison with previous studies

Earlier literature has reported varicella zoster virus (VZV)-associated central nervous system (CNS) vasculopathy in children presenting with ischemic strokes rather than hemorrhages. The VIPS II study, conducted by Fullerton et al. (6), found evidence of VZV reactivation in a substantial number of children with arterial ischemic stroke, strengthening the role of the virus in post-infectious cerebrovascular syndrome through mechanisms of vasculopathy. A parallel systemic review and meta-analysis by Lu et al. (7) demonstrated a significantly higher risk of stroke after VZV infection, primarily ischemic, with hemorrhagic strokes being rare and less well-defined in children. Clarke et al. (8) further emphasized that, although viral infections have emerged as significant causes of ischemic stroke due to vascular inflammation, hemorrhagic presentations are rare.

Unlike ischemia-dominant presentations, our case was distinctly characterized by an isolated cerebellar hemorrhagic event due to vasculitis, as diagnosed through MRI, MR angiography, and MR spectroscopy, in a previously healthy, immunocompetent child. Previously reported hemorrhagic strokes in children following VZV infection have typically occurred in immunocompromised children or have involved multifocal ischemic lesions with small hemorrhagic components (9, 10). The uniqueness of our case is the lack of a predisposing vascular disease or immunosuppression, with the patient instead presenting a single, radiologically verified cerebellar vasculitic hemorrhage occurring after an uneventful case of varicella disease—a manifestation that is not commonly reported in the current literature.

Furthermore, Grose et al. (11) elaborated on VZV neurotropism, with the virus’s latency and reactivation in cranial nerve ganglia, especially the trigeminal ganglion, being implicated in cerebrovascular sequelae. However, the majority of these occurrences have been attributed to ischemic strokes, with hemorrhagic transformation being extremely uncommon. This case thus provides useful information about the spectrum of VZV-induced cerebrovascular disease, building on the growing body of evidence supporting viral infections as underappreciated causes of pediatric stroke and emphasizing the importance of increased clinical awareness (8, 9).

In this case, the patient developed a vasculitic hemorrhage, one of the serious late complications of a recent varicella zoster virus (VZV) infection. It is reasonable to assume that the small parietal lobe hemorrhage detected originated from vasculitic inflammation of the cerebral arteries. Magnetic resonance angiography (MRA), performed to assess for localized cerebral vasculitis, provided further evidence of vascular inflammation secondary to VZV. Given that the patient is a previously healthy child, it is noteworthy that VZV vasculitis is rarely observed in individuals with intact immune systems and is more commonly associated with immunosuppressed conditions (8). Neurological involvement often occurs within the first few weeks of the primary infection or during reactivation, underscoring the importance of timely diagnosis and appropriate management (9).

VZV can be managed with antiviral medications, while corticosteroids are administered to decrease inflammation and limit further blood vessel damage. Anticoagulants, including aspirin, are also used to prevent thrombotic complications when vasculitis is present (10).

Regarding the vasculitic and hemorrhagic complications of VZV, the severity of vascular involvement and the timing of treatment initiation are key determinants of the outcomes. The timing of antiviral treatment and the stage of the disease at which early intervention occurs are of paramount importance in preventing chronic neurological sequelae (11). In this case, treatment was effective for the young child, as there was no further progression of hemorrhage or vasculitis. However, long-term follow-up is necessary to monitor for any possible recurrence or late neurological sequelae.

## Conclusion

This example portrays the great possibilities for vascular complications in the pediatric population after varicella infection, even in the absence of extrapersonal predisposing factors. Although VZV vasculitis is uncommon, its presence must be considered in any child showing new neurological manifestations with a history of a recent or remote VZV infection. Above all, vascular complications may rarely occur either during active viral replication or in a post-infectious, immune-mediated fashion. In the presence of an active infection, antivirals (e.g., acyclovir) are paramount in limiting viral replication and thus minimizing direct vascular damage. In contrast, for post-infectious vasculitis, when viruses cease replication, the anti-inflammatory approach (e.g., corticosteroids) assumes major significance in controlling vascular inflammation that is immune-mediated. Early identification and treatment tailored to the underlying pathophysiology are the most effective ways to improve neurological outcomes. A greater understanding of the triggering mechanisms behind both active and post-infectious VZV-related vasculitis would allow for more focused prevention and treatment options.

## Data Availability

The original contributions presented in the study are included in the article/supplementary material, further inquiries can be directed to the corresponding author.
